# Structural and functional analysis of Utp24, an endonuclease for processing 18S ribosomal RNA

**DOI:** 10.1371/journal.pone.0195723

**Published:** 2018-04-11

**Authors:** Weidong An, Yifei Du, Keqiong Ye

**Affiliations:** 1 College of Biological Sciences, China Agricultural University, Beijing, China; 2 National Institute of Biological Sciences, Beijing, China; 3 Key Laboratory of RNA Biology, CAS Center for Excellence in Biomacromolecules, Institute of Biophysics, Chinese Academy of Sciences, Beijing, China; 4 University of Chinese Academy of Sciences, Beijing, China; Scripps Research Institute, UNITED STATES

## Abstract

The precursor ribosomal RNA is processed by multiple steps of nucleolytic cleavage to generate mature rRNAs. Utp24 is a PIN domain endonuclease in the early 90S precursor of small ribosomal subunit and is proposed to cleave at sites A1 and A2 of pre-rRNA. Here we determine the crystal structure of Utp24 from *Schizosaccharomyces pombe* at 2.1 angstrom resolution. Utp24 structurally resembles the ribosome assembly factor Utp23 and both contain a Zn-finger motif. Functional analysis in *Saccharomyces cerevisiae* shows that depletion of Utp24 disturbs the assembly of 90S and abolishes cleavage at sites A0, A1 and A2. The 90S assembled with inactivated Utp24 is arrested at a post-A0-cleavage state and contains enriched nuclear exosome for degradation of 5' ETS. Despite of high sequence conservation, Utp24 from other organisms is unable to form an active 90S in *S*. *cerevisiae*, suggesting that Utp24 needs to be precisely positioned in 90S. Our study provides biochemical and structural insight into the role of Utp24 in 90S assembly and activity.

## Introduction

Assembly of yeast ribosome requires processing and modification of rRNAs and association of 79 ribosomal proteins [1, 2]. This process begins with the transcription of a 35S precursor rRNA (pre-rRNA) that encodes 18S, 5.8S and 25S rRNAs and the external (5' ETS and 3' ETS) and internal (ITS1 and ITS2) transcribed spacers. The four spacers need to be removed by a series of endo- and exo-nucleolytic activities in the context of various pre-ribosomal particles [3].

Early processing of 18S rRNA occurs in the nucleolus within the 90S pre-ribosome or the small subunit processome [4, 5], which is the early assembly intermediate of small ribosomal subunits. The 90S pre-ribosome is co-transcriptionally assembled from ~70 non-ribosomal assembly factors (AFs), U3, U14, snR30 and snR10 snoRNAs and ~ 20 ribosomal proteins in a stepwise and dynamic manner [6–8]. At the final stage of 90S formation, the U14 and snR30 snoRNAs and a dozen labile AFs recruited by the 18S region are released, resulting in a fully assembled 90S [6, 9]. The cryo-EM structures of 90S have been recently determined, revealing that the nascent 40S ribosome is assembled into several isolated subdomains and stabilized by numerous AFs [10–14].

The pre-rRNA is sequentially cleaved at the A0 and A1 sites of the 5' ETS and at the A2 site of the ITS1. Consequently, the 90S is transformed into a pre-40S ribosome that contains a 20S pre-rRNA [15]. The 20S pre-rRNA is further processed at the D site in the cytoplasm to give rise to the mature 18S rRNA. In an alternative pathway, the 35S pre-rRNA is first cleaved at the A3 site in the ITS1, yielding a 23S intermediate for small subunits. The 23S pre-rRNA is also considered as a non-functional intermediate that strongly accumulates under stress conditions [16]. It can be targeted by an RNA surveillance system that involves oligoadenylation by the TRAMP complex and degradation by the nuclear exosome [17].

The PIN (PilT N-terminus) domain is a small domain with ~ 130 amino acid residues and is widely distributed in all three kingdoms of life [18]. It typically functions as an endonuclease in degradation and processing of various RNAs. The activity of PIN domain depends on four conserved acidic residues and the presence of Mn^2+^ or Mg^2+^ ion [19, 20]. Three PIN domain proteins, Utp24, Utp23 and Nob1, have been found to be required for 18S rRNA processing. Nob1 cleaves the D site, generating the 3' end of 18S rRNA [21]. Utp23 contains a degenerated PIN domain and functions together with snR30 snoRNA in the assembly of 90S [22–25].

Utp24 is the endonuclease proposed to cleave the A1 and A2 sites. Depletion of Utp24, aka Fcf1 (Faf1 copurifying factor 1), inhibits cleavage at sites A0, A1 and A2 in yeast [24, 26], whereas mutations of the active site of Utp24 specifically block cleavage at sites A1 and A2, but not at site A0 [24]. This suggests that Utp24 plays both a structural and catalytic role in 90S. In humans, inactivation of UTP24 similarly inhibits cleavage at the equivalent 1 and 2a sites [27, 28]. Structurally, Utp24 crosslinks with the 5' end of 18S [27] and is positioned near the A1 site in the cryo-EM structures of 90S [10–14]. These data strongly support that Utp24 is the endonuclease for site A1.

Genetic analyses also show that the activity of Utp24 is required for processing site A2 [24, 27, 28]. Recombinant Utp24 proteins demonstrate in vitro cleavage activity on site A2, suggesting that Utp24 is also the nuclease for site A2 [27]. The specificity of cleavage critically depended on Mn^2+^ at reaction conditions and was lost in the presence of Mg^2+^ [27]. Rcl1, a protein homologous to RNA 3'-terminal phosphate cyclase, was previously proposed to cleave site A2 [29]. However, the nuclease activity and the active site of Rcl1 were not confirmed [27, 30].

Although the defects in pre-rRNA processing caused by Utp24 depletion and mutation have been well established, the molecular basis of the phenotype is unclear. Recently, Utp24 was modeled in the cryo-EM structures of *Saccharomyces cerevisiae* and *Chaetomium thermophilum* 90S [11, 13]. In this study, we determined a crystal structure of the PIN domain of Utp24 from *Schizosaccharomyces pombe*. We also analyzed how depletion and mutation of Utp24 affect the assembly and rRNA processing activity of 90S in *S*. *cerevisiae*. Our study provides biochemical and structural insight into the role of Utp24 in 90S assembly and activity.

## Results

### Crystal structure of Utp24

We expressed and purified recombinant Utp24 proteins from several organisms and succeeded in crystalizing Utp24 from *S*. *pombe* (spUtp24). The crystal belongs to space group C222_1_ and contains one molecule in the asymmetric unit. The structure was determined with molecular replacement using the Utp23 structure as search model [22] and refined to an R_free_/R_work_ of 0.287/0.222 at 2.14 Å resolution (Table 1).

**Table 1:** Statistics of data collection and structural refinement.

Data collection	spUtp24
Beamline	SSRF BL17U
Wavelength (Å)	0.979135
Space group	C222_1_
Unit cell dimensions	
a, b, c (Å)	48.7, 73.3, 74.3
α, β, γ (°)	90.0, 90.0, 90.0
Resolution (Å)	50–2.13(2.17–2.13)
Unique reflections	7270 (284)
Completeness (%)	94.5 (73.6)
Redundancy	12.1 (7.3)
<I/σ(I)>	18.9 (1.9)
Rmerge	0.184 (>1)
CC1/2	(0.795)
Refinement	
Resolution (Å)	40.6–2.13(2.3–2.13)
Number of reflections	6721 (992)
Number of atoms	1088
Protein	1072
Ion	2
Water	14
R-work	0.222(0.283)
R-free	0.287(0.397)
Mean B factor (Å^2^)	33.97
RMSD bond length (Å)	0.008
RMSD bond angles (°)	0.903
Ramachandran plot (%)	
Favored	94.66
Allowed	3.82
Outliers	1.53

Numbers in parenthesis are for the highest resolution shell

**Fig 1:**
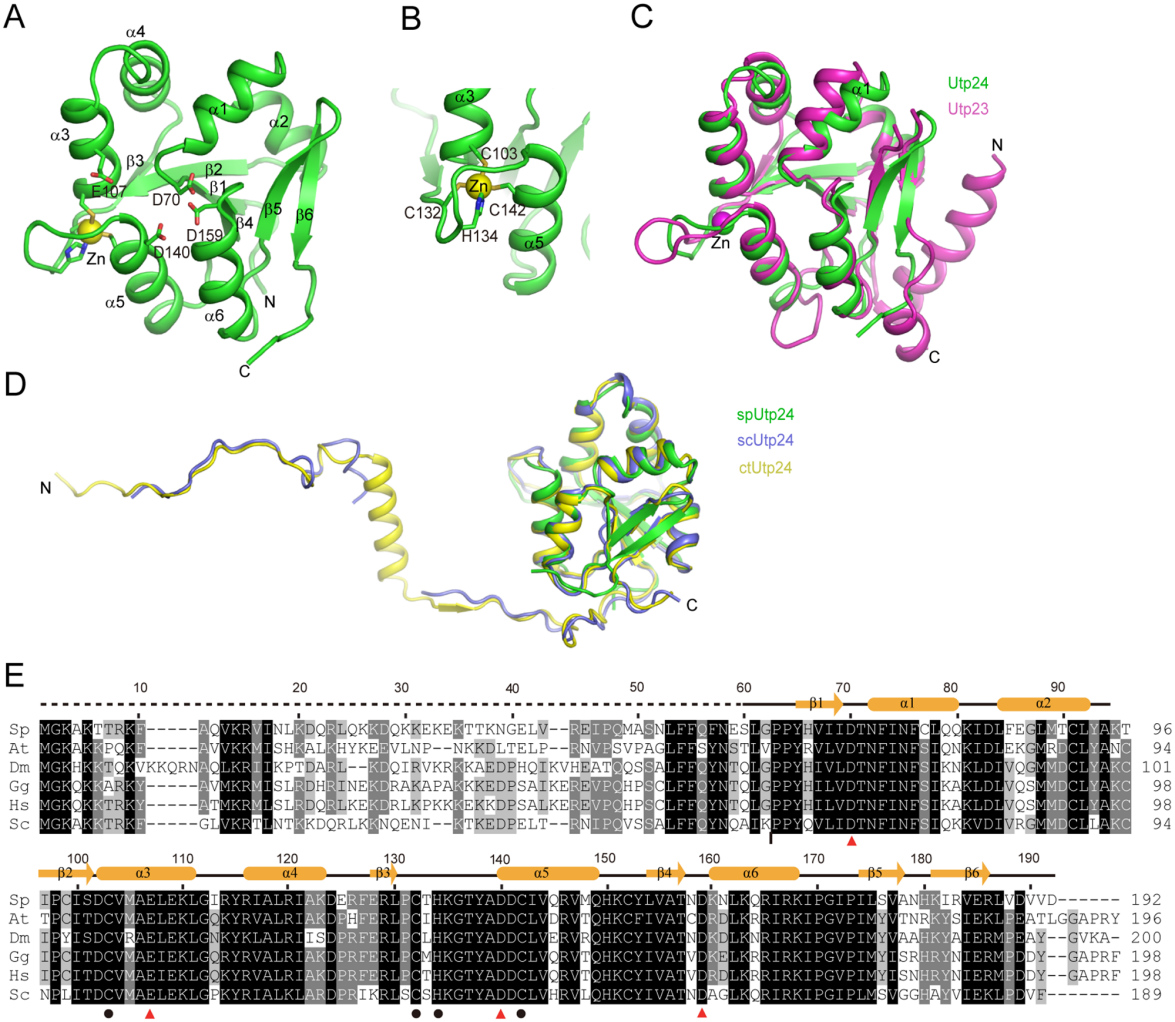
Structure of *S*. *pombe* Utp24. (A) Ribbon representation of spUtp24 structure. The secondary structures and the N- and C-termini are labeled. The catalytic residues and Zn-coordinating residues are shown as sticks and the bound Zn ion as sphere. (B) Structure of the Zn-finger motif. (C) Structural alignment of Utp24 and Utp23 (PDB code 4MJ7). (D) Alignment of crystal structure of spUtp24 with cryo-EM structures of scUtp24 (PDB code 5WLC) and ctUtp24 (PDB code 5OQL). (E) Multiple sequence alignment of Utp24. The Utp24 sequences from *S*. *cerevisiae* (Sc), *Homo sapiens* (Hs), *Gallus gallus* (Gg), *Drosophila melanogaster* (Dm), *Arabidopsis thaliana* (At) and *S*. *pombe* (Sp) are aligned. Residues conserved in at least 100%, 80% and 60% of these sequences are shaded in black, gray and light gray, respectively. The secondary structure elements observed in the crystal structure are shown on the top. The dotted line denotes the region not resolved in crystal structure. At the bottom of the alignment, the catalytic and Zn-coordinating residues are denoted by triangles and circles, respectively. A vertical bar marks the joining point of scUtp24 and hUTP24 in the chimera protein.

Although the full-length protein of spUtp24 was used in crystallization, the crystal structure contains only the PIN domain (residues 60–192). The N-terminal region was not resolved likely due to flexibility or degradation. The structure adopts a compact three-layer fold with a mixed six-strand β-sheet packed by helices α1-α4 at one side and helices α5 and α6 at the other side (Fig 1A). A zinc ion is coordinated with Cys103, Cys132, His134 and Cys142 (Fig 1B), which are invariant in Utp24 orthologues (Fig 1E). PIN domains contain commonly four acidic residues required for catalysis. These residues, Asp70, Glu107, Asp140 and Asp159 in spUtp24, are clustered at the N-termini of helices α1, α5 and α6 and the middle of helix α3.

The structures of Utp24 and Utp23 are superimposable with a root mean square deviation (RMSD) of 1.06 Å over 92 Cα pairs (Fig 1C). Both Utp24 and Utp23 contain a CCHC-type Zn-finger motif. Utp23 has an extra α-helix at the N-terminus, which is highly basic and essential for Utp23 function [22]. In addition, the conformation of a few loops and the orientation of helix α1 are varied between the two structures.

The spUtp24 structure is well aligned to the recently determined cryo-EM structures of *S*. *cerevisiae* and *C*. *thermophilum* Utp24 (scUtp24 and ctUtp24) in 90S with RMSD = 0.86–0.98 Å (Fig 1D) [11, 13]. However, the C-terminal tail of Utp24 (residues 184–189 of scUtp24) changes its orientation upon association with 90S. The cryo-EM structures also reveal that the N-terminal tail of Utp24 adopts an extended conformation and contacts multiple components in 90S [11, 13].

### Functional analysis of Utp24

**Fig 2:**
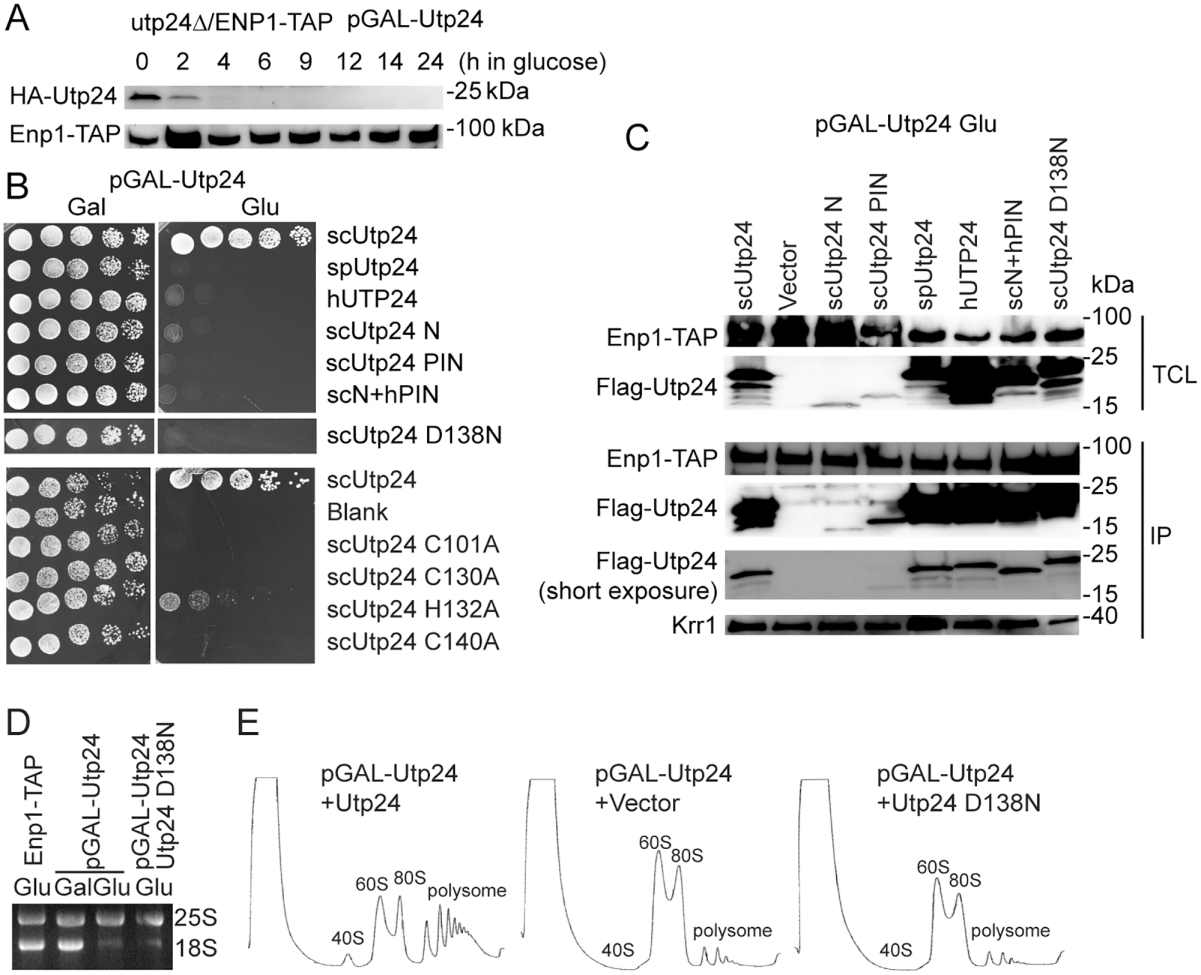
Functional assay of Utp24 in *S*. *cerevisiae*. (A) Depletion of Utp24 in the Utp24 shuffle strain after transfer to glucose medium. Total proteins were resolved with SDS-PAGE and analyzed with Western blot using anti-HA and PAP antibodies. The PAP antibody recognizes protein A in the TAP-tag. (B) Growth assay. The utp24Δ/ENP1-TAP strain complemented by a *URA3* plasmid expressing wild-type Utp24 under control of a *GAL* promoter was transformed with a *LEU2* plasmid expressing WT or mutant Utp24, Utp24 from human or *S*. *pombe*, or a chimera protein containing the N-terminal residues 1–60 (N) of scUtp24 and the PIN domain (residues 65–198) of hUTP24. Five folds serial dilutions of cells were spotted on Ura- and Leu-deficient SC medium containing galactose (Gal) or glucose (Glu) and incubated at 30°C. (C) Association of Utp24 mutants with 90S. The strains described in (B) were grown in glucose for 14 h. Total cell lysates (TCL) and immunoprecipitations (IP) of IgG coated beads were analyzed with Western blot using PAP, anti-Flag and anti-Krr1 antibodies. Positions of molecular weight makers are indicated. (D) An agarose gel showing rRNA stained by ethidium bromide. Total RNAs were extracted from ENP1-TAP strain, Utp24 shuffle strain grown in YPG and then in YPD, or Utp24 shuffle strain expressing the D138N mutant of Utp24 grown in YPD. (E) Ribosome profile. Extracts of Utp24 shuffle strains transformed with a plasmid expressed wild-type or D138N mutant Utp24 or an empty plasmid grown in glucose were fractionated on 7%-50% sucrose gradients.

To investigate the function of Utp24 in *S*. *cerevisiae*, the Utp24 shuffle strain was constructed in a strain expressing tandem affinity purification (TAP)-tagged Enp1 by disrupting the chromosomal *UTP24* gene and expressing HA-tagged Utp24 under control of a *GAL* promoter from a *URA3* plasmid. Enp1-TAP can be utilized to affinity purify 90S and pre-40S pre-ribosomes [15]. The wild-type Utp24 protein was efficiently depleted when the strain was grown in glucose (Fig 2A). Flag-tagged Utp24 mutants were expressed from a *LEU* plasmid and assessed for function (Fig 2B–2E). Mutation of the catalytic residue Asp138 to asparagine (D138N) abolished the yeast growth (Fig 2B). Depletion or inactivation of Utp24 significantly inhibited the production of 18S rRNA and 40S subunit (Fig 2D and 2E). These results confirm that Utp24 and its nuclease activity are essential for 18S rRNA processing and cell growth [24, 28].

To examine the functional importance of the Zn-finger motif, the four Zn-coordinating residues were mutated to alanine (Fig 2B). Mutation of each of three cysteine ligands caused lethality. Mutation of the histidine ligand inhibited the growth, but was not lethal. Previous mutational analysis of Utp23 also found the essential role of cysteine ligands and the non-essential role of histidine ligand [22]. Hence, the characteristic Zn-finger motif is an essential element of the PIN domain of both Utp24 and Utp23.

In the crystal structure, the N-terminal tail of spUtp24 was not resolved. The PIN domain or the N-terminal tail alone did not support the yeast growth, suggesting that both parts are indispensable. However, this phenotype may be partially caused by the low expression level of the two fragments (Fig 2C).

Utp24 is an extremely conserved protein with 58% identity and 74% similarity between the human and *S*. *cerevisiae* sequences, 60% identity and 80% similarity between the *S*. *pombe* and *S*. *cerevisiae* sequences (Fig 1E). Notably, Utp24 from human (hUTP24) and *S*. *pombe* were unable to substitute the function of scUtp24 (Fig 2B). hUTP24 has been previously shown not to be functional in yeast [28]. The PIN domain is extremely conserved in Utp24, but the N-terminal tail is more variable (Fig 1E). To test whether the N-terminal tail confers any species specificity of Utp24, we constructed a chimera protein that contains the N-terminal sequence of scUtp24 and the PIN domain of hUTP24. The chimera protein was not functional either, suggesting that Utp24 is highly specific to each organism.

To examine whether these Utp24 variants can be assembled into the *S*. *cerevisiae* 90S, we immunoprecipitated pre-ribosomes with TAP-tagged Enp1, which is a component of both 90S and pre-40S pre-ribosomes [15], and examined the binding of Utp24 by Western blot (Fig 2C). The spUtp24, hUTP24 and chimera protein were all co-immunoprecipitated as efficiently as scUtp24. Hence, these Utp24 variants were assembled into 90S, but did not yield functional 90S pre-ribosomes. The association of Krr1, an AF bound to the central domain of 18S rRNA, was not affected by Utp24 depletion or mutation (Fig 2C).

### Contribution of Utp24 to pre-rRNA processing

**Fig 3:**
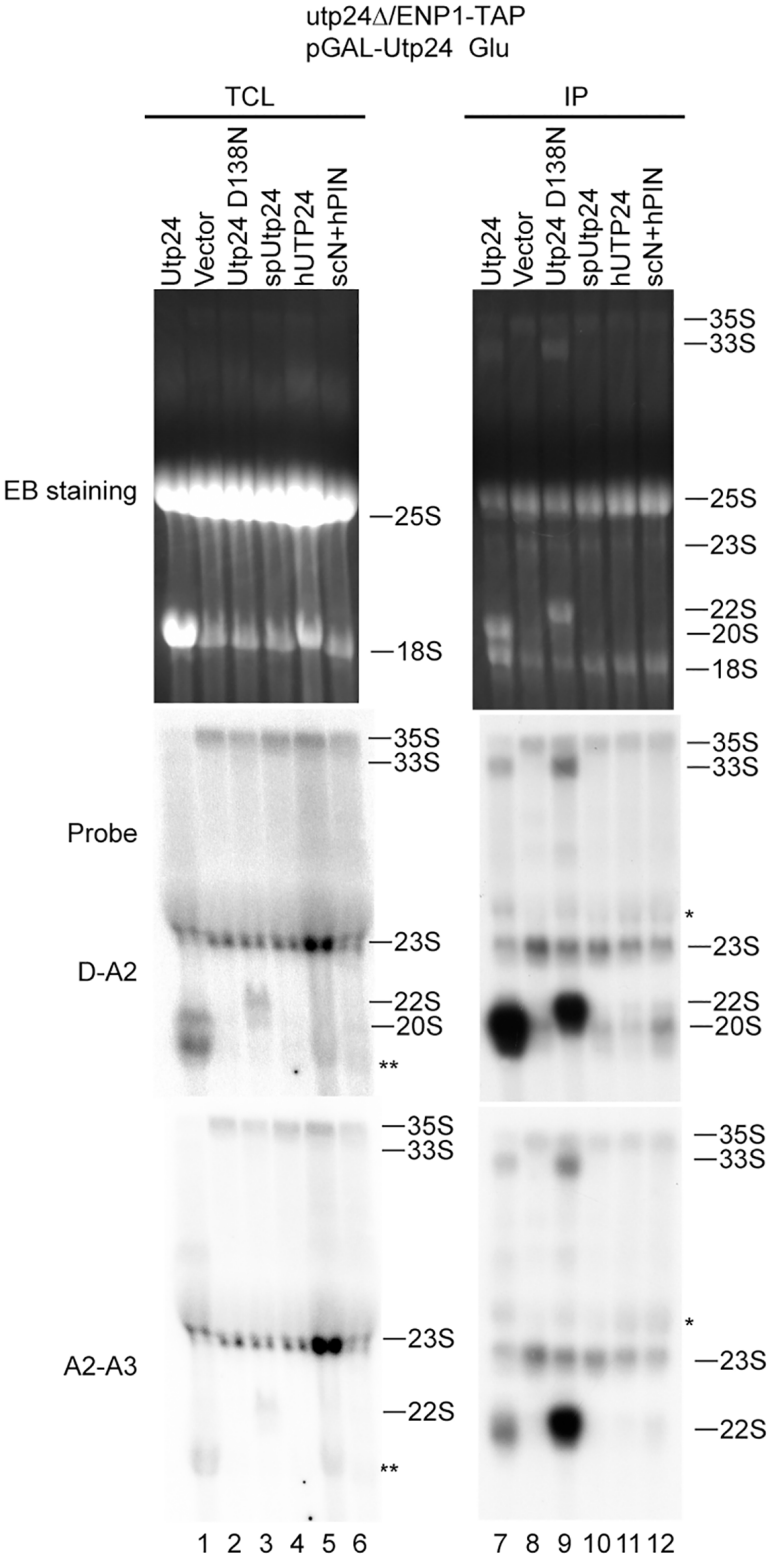
Northern blot analysis of pre-rRNA processing intermediates. Utp24 shuffle strains transformed with a plasmid encoding wild-type or variant Utp24 were grown YPG and then shifted to YPD for 14 h. RNAs extracted from total cell lysate (TCL) and IgG immunoprecipitations (IP) were resolved in 1.2% agarose-formaldehyde gels and strained by ethidium bromide (EB). RNAs were transferred to Hybond N^+^ membranes, hybridized against ^32^P-labeled probes and visualized by autoradiography. Asterisk and double asterisk denote non-specific bands of 25S and 18S rRNA, respectively.

To examine how depletion and mutation of Utp24 affects pre-rRNA processing (S1 Fig), the pre-rRNA processing intermediates in total RNA were analyzed with Northern blot (Fig 3). Depletion of Utp24 led to an accumulation of 35S and 23S pre-rRNAs and blocked production of 20S pre-rRNA (lanes 1 and 2). Expression of the inactive D138N mutant of Utp24 caused a strong accumulation of 22S pre-rRNA (lane 3). These results are consistent with the previous studies [24]. We also analyzed the pre-rRNAs present in the pre-ribosomes purified via Enp1-TAP. In the presence of wild-type Utp24, Enp1-TAP co-purified a large amount of 20S pre-rRNA as well as other various processing intermediates: 35S, 33S, 23S and 22S pre-rRNAs (lane 7). Much of the 20S pre-rRNA should originate from the abundant pre-40S ribosome. Depletion of Utp24 led to a strong accumulation of 35S and 23S pre-rRNAs and a dramatic decrease of 33S, 22S and 20S pre-rRNAs (lane 8), indicating that cleavage at the A0, A1 and A2 sites was blocked in the ΔUtp24 90S. In the presence of the D138N mutant, the 22S pre-rRNA strongly accumulated and the 20S pre-rRNA was absent (lane 9), indicating that the nuclease activity of Utp24 is required for the A1 and A2 cleavage, but not for the A0 cleavage. Overall, the northern blot results from purified pre-ribosomes are consistent with and better resolved than those from total RNA.

As spUtp24, hUTP24 and the chimera protein can be assembled into the *S*. *cerevisiae* 90S, we asked whether they are functional in pre-rRNA processing. None of these heterologous Utp24 proteins restored the level of 20S pre-rRNA in total RNAs and Enp1-TAP particles (lanes 4–6 and 10–12). The 90S particles assembled with hUTP24 and the chimera Utp24, but not spUtp24, contained slightly increased levels of 22S pre-rRNA compared to the ΔUtp24 90S, suggesting that the A0 cleavage was partially restored (lanes 10–12).

### Contribution of Utp24 to 90S assembly

**Fig 4:**
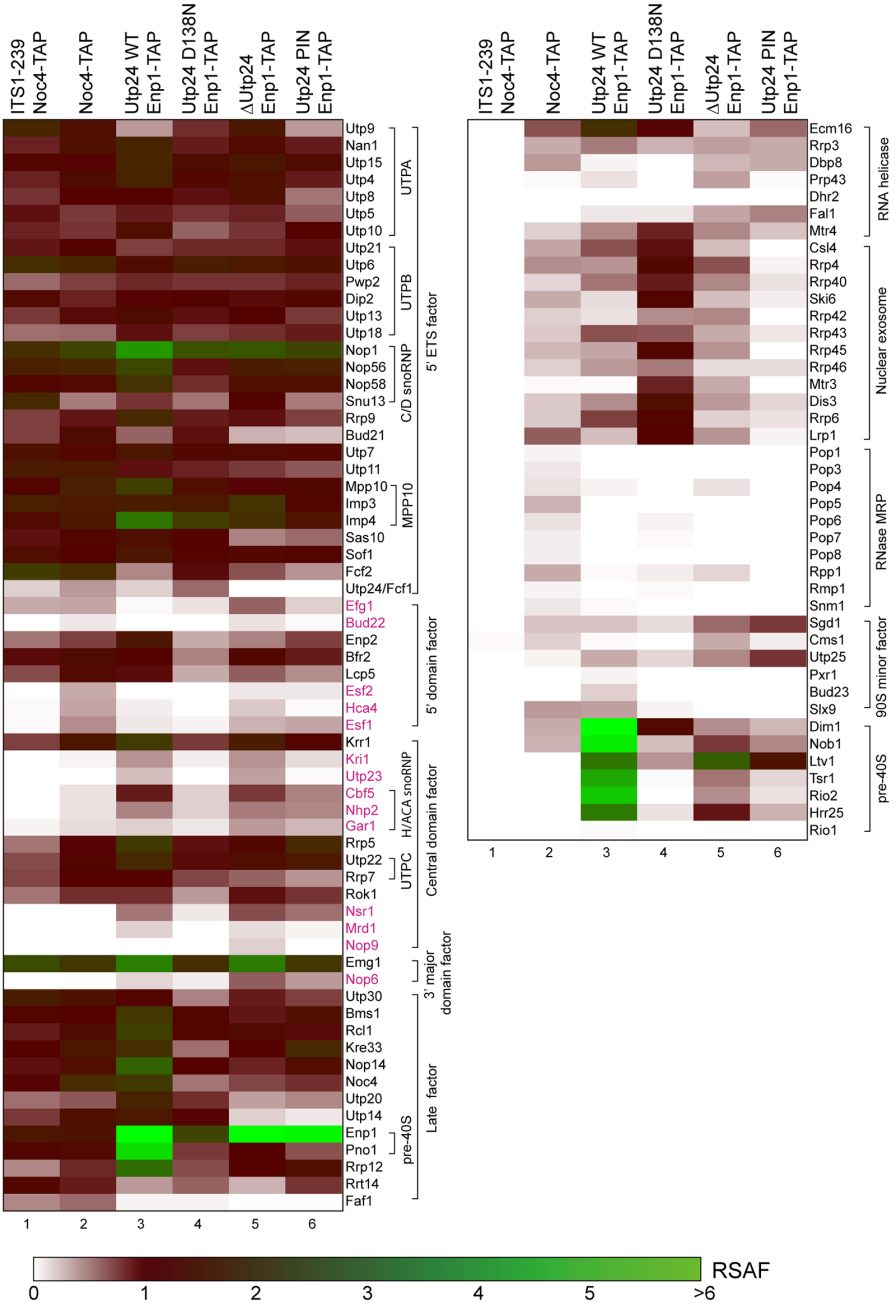
Heatmap of AFs in pre-ribosomes. Utp24 shuffle stains alone or expressing WT or mutant Utp24 were grown in YPG and shifted to YPD for 14 h to deplete wild type Utp24. The first two samples were previously reported and included for comparison [6]. The first sample (ITS1-239/Noc4-TAP) was purified via a plasmid-derived pre-18S fragment ending at position 293 of ITS1 and Noc4-TAP and stands for a fully assembled 90S particle that lacks labile AFs and contains an unprocessed 5' ETS. The second Noc4-TAP sample was purified in two steps. All other samples were purified with single step of IgG immunoprecipitation. Proteins are color-coded according to their relative spectral abundance factor (RSAF) values relative to the UTPB proteins. Proteins in magenta are labile AFs diminished in fully assembled 90S particles.

Utp24 is a stable component of 90S and deeply buried in the 90S structure [10–14]. We asked how depletion and mutation of Utp24 affect the assembly of 90S. We purified small subunit pre-ribosomes via TAP-tagged Enp1 and analyzed the associated proteins with semi-quantitative mass spectrometry [6, 31]. In the presence of wild-type Utp24, Enp1-TAP co-purified majority of known 90S AFs and excessive late pre-40S AFs, including Pno1, Dim1, Nob1, Ltv1, Tsr1, Rio2 and Hrr25 (Fig 4, lane 3). This is expected since Enp1 is present in both the 90S and pre-40S preribosomes [15].

No peptide of Utp24 was detected in the Enp1-TAP particles when expression of Utp24 was repressed in glucose (lane 5). This confirmed that Utp24 was effectively depleted. We also analyzed Enp1-TAP particle in the presence of the PIN domain fragment of Utp24 but detected no Utp24 peptide in the purified pre-ribosomes (lane 6). This may be due to the low expression level and/or weak binding of the PIN domain fragment (Fig 2C). The sample with depleted Utp24 contained increased level of Efg1 compared to other samples. The sample with truncated Utp24 showed reduced binding of Hca4, Utp23 and nuclear exosome, compared to the sample with depleted Utp24. Otherwise, the two samples with depleted and truncated Utp24 were largely equivalent. Except for Ltv1, the late pre-40S AFs were dramatically reduced, indicating that the 90S failed to develop into the pre-40S in the absence of Utp24. Ltv1 was enriched because it formed a subcomplex with Enp1 and Rps3 [32, 33]. Most 90S AFs exhibited similar abundance between the ΔUtp24 and wild-type 90S particles, but some AFs showed greater than 2-fold changes. Most dramatically, binding of Utp14 in 90S particles in the absence of Utp24 was reduced to approximately 10% compared to particles containing wild type Utp24. Utp14 is an essential AF associated with the 90S pre-ribosome [5, 34]. In addition, the levels of Bud21 and Sas10 were decreased by nearly half. Several minor and labile AFs, including Nop6, Fal1, Sgd1 and Utp25, were more enriched, suggesting that the ΔUtp24 90S was arrested in early assembly states.

In the presence of inactivated Utp24, the protein profile of Enp1-TAP particle was similar to that of Noc4-TAP particle (Fig 4, lane 4). Noc4 is specific to 90S and co-purified little late pre-40S AFs. This indicates that the 90S was normally assembled, but cannot progress into pre-40S in the absence of Utp24 activity. In addition, the nuclear exosome and Mtr4 that are responsible for degrading the cleaved 5' ETS were strongly enriched [35, 36], which apparently acts as a response to the increased A0 cleavage of pre-rRNA in 90S with inactivated Utp24.

## Discussion

We have determined a high resolution structure of the PIN domain of Utp24. The structure shows close resemblance with the structure of the degenerated PIN domain of Utp23 [22]. Both structures share a characteristic Zn-finger motif that is not present in other PIN domains [18]. The strong structural similarity suggests that the two PIN domain proteins, both involved in early 18S rRNA processing, have a close evolutionary relationship. Nevertheless, Utp23 and Utp24 play distinct roles in the assembly and function of 90S. Utp23 apparently lacks a nuclease activity. Utp23 and its binding partner snR30 associate only with the assembly intermediate of 90S [6, 22, 23, 25], whereas Utp24 is a stable component of 90S pre-ribosome. Utp23 and Utp24 each possess extra structural elements outside the core PIN domain to fulfill their distinct functions. The function of Utp23 critically depends on two Utp23 specific elements: an N-terminal α-helix and a C-terminal tail [22]. Utp24 has an N-terminal tail that interacts extensively with the 90S structure [11, 13]. The functional importance of the N-terminal tail of Utp24 cannot be assessed in our study since Utp24 is poorly expressed without the N-terminal tail.

As Utp24 functions within the 90S, the phenotype of Utp24 depletion or mutation on pre-rRNA processing is based on the subsequent changes in the structure and processing activity of 90S. To probe the structural role of Utp24, we analyzed the pre-rRNA and protein components of 90S after depleting or mutating Utp24. Utp24 forms an extensive interaction network in 90S [10–14] and is recruited at early stage during the ordered assembly pathway of 90S [6], but removal of Utp24 does not grossly affect the composition of 90S. This is in contrast with deletion of UTPA proteins, which are the first AFs bound to the pre-rRNA and required for the association of many downstream factors [37, 38]. Nevertheless, the absence of Utp24 dramatically reduces the association of Utp14. Utp14 is essential for 18S rRNA processing and interacts with Dhr1/Ecm16, an RNA helicase proposed to release the U3 snoRNA [5, 34]. Utp14 does not directly contact Utp24 in the 90S structure [11, 13], suggesting that lack of Utp24 disturbs the assembly of 90S in a complicated manner. These compositional changes would subsequently block the co-transcriptional formation of the compact structure of 90S on nascent pre-rRNA [24], leading to defective processing at the A0, A1 and A2 sites.

When Utp24 is inactivated, the 90S is normally assembled and arrested at a post-A0-cleavage state. The resultant 90S already cleaves majority of pre-rRNA at the A0 site, but cannot process the A1 and A2 sites due to the lack of Utp24 activity, leading a strong accumulation of 22S rRNA. As a response to the enhanced A0 cleavage, more nuclear exosome appears to be recruited to degrade the cleaved 5' ETS. Moreover, the 5' EST fragment may be inaccessible to the exosome in the mutant 90S. Consequently, more exosome may get trapped in the 90S.

The pre-rRNA is processed at sites A0, A1 and A2 within the 90S pre-ribosome. In the cryo-EM structures of 90S, the A1 site is intact and not loaded to the active site of Utp24, indicating that these determined structures represent pre-A1-cleavage states [10–14]. The processing of A0 and A2 sites are poorly understood at present. No nuclease has been identified for A0 cleavage and the nuclease for site A2 is debated. The A0 and A2 sites are not visible in the determined cryo-EM structures of 90S, suggesting that they are located outside of the 90S core structure. Nevertheless, defective 90S structures caused by, for example, depletion of Utp24 blocks processing of site A0, suggesting that the A0 cleavage also requires a properly assembled 90S structure.

The Utp24 proteins from *S*. *pombe* and human share high degree of sequence similarity with *S*. *cerevisiae* Utp24. These heterologous Utp24 proteins can be assembled into the *S*. *cerevisiae* 90S pre-ribosome, but the resultant 90S preribosomes are defective in rRNA processing. This suggests that precise placement of Utp24 in 90S is essential and may depend on some species-specific interactions.

## Materials and methods

### Gene cloning and protein purification

The full-length spUtp24 gene was PCR-amplified from a cDNA library of *S*. *pombe* and cloned into a modified pET28a plasmid with the In-fusion method. The spUtp24 protein was fused to an N-terminal His_6_-Smt3-tag. The protein was expressed in *E*. *coli* Rosseta (DE3) cells in LB medium with 50 μg/ml kanamycin. After the cells were cultured at 37 °C to OD_600_ of 0.8–1.0, the protein expression was induced with 0.5 mM isopropyl β-D-1-thiogalactopyranoside at 18 °C for 18 h.

The cells were collected by centrifugation, resuspended in lysis buffer (50 mM Tris-Cl, pH 8.0, 400 mM NaCl) and lysed with a JN-3000 cell disruptor (JNBIO). The cell lysate was clarified by centrifugation with 18000 rpm at 4 °C for 1 h. The supernatant was filtered with 0.45 μm filters and loaded on a 5-ml HisTrap column (GE Healthcare). The column was washed with 50 mM imidazole in lysis buffer and eluted with 500 mM imidazole in lysis buffer. The fractions containing the target protein were pooled and incubated with Ulp1 protease at 4 °C for 1 h to remove the His_6_-Smt3 tag. The sample was loaded onto a heparin column and eluted with a linear gradient of 0.4–1 M KCl in 20 mM HEPES-KOH, pH 7.6. The peak fractions were collected, concentrated and loaded onto a HiLoad 16/60 Superdex 200 column (GE Healthcare) in buffer (10 mM Tris-Cl pH 8.0 and 400mM NaCl). The target protein was concentrated and stored at -80 °C.

### Crystallization and structure determination

The full length spUtp24 protein was crystallized at 20 °C by the sitting-drop vapor diffusion method by mixing 1μl of protein sample (10 mg/ml in 10 mM Tris-Cl, pH 8.0 and 400 mM NaCl) with 1μl of reservoir solution (0.2 M calcium acetate and 20% polyethylene glycol 3350). Crystals appeared as cluster after 1 month. Single crystals were separated, cryo-protected in 15% glycerol prepared in the reservoir solution, and flash frozen in liquid nitrogen.

Diffraction data were collected at the Shanghai Synchrotron Radiation Facility at beamline BL17U and processed using HKL2000 [39]. The structure was determined with molecular replacement using the crystal structure of Utp23 as search model in PHENIX [22]. The model was built in COOT and refined in PHENIX [40, 41].

The crystal belongs to space group C2221 and has one spUtp24 molecule in the asymmetric unit. The final model contains residues 60–192 of spUtp24, one Zn^2+^ ion, one Ca^2+^ ion and 14 water molecules. The Ca^2+^ ion is bound at the crystallographic interface of two molecules and is modeled as half occupancy. The Ca^2+^ ion is coordinated by Glu128 and His66 of both molecules and not located at the active site of Utp24.

### Plasmids and yeast strains

Yeast cells were manipulated according to standard protocols and cultured in synthetic complete (SC) medium with glucose or galactose, appropriate SC dropout medium, YPD (1% yeast extract, 2% peptone, 2% glucose) and YPG (1% yeast extract, 2% peptone, 2% galactose). All strains are derived from BY4741 and listed in S1 Table. The *UTP24* gene was cloned into a *URA3* pRS416 plasmid under a *GAL* promoter and a HA tag with the Transfer-PCR (TPCR) method [42], yielding the plasmid pRS416-GAL-HA-UTP24. We failed to directly replace the promoter of the chromosomal *UTP24* gene with a *GAL* promoter. To make a *UTP24* shuffle strain, the ENP1-TAP strain was first transformed with the pRS416-GAL-HA-UTP24 plasmid. The chromosomal *UTP24* gene was then deleted by homologous recombination with a PCR product containing the *natNT2* gene from plasmid pYM-N28 [43].

The *UTP24* genes were PCR amplified from *S*. *cerevisiae* genomic DNA or cDNA libraries of *S*. *pombe* and *H*. *sapiens* and cloned into a *LEU2* pRS415 plasmid downstream of a *GPD* promoter and a 3xFLAG tag with the In-fusion method. Point mutation and deletion were introduced with the QuikChange method. To construct the chimeric Utp24 protein, the DNA sequences encoding residues 1–60 of scUtp24 were amplified from pRS415-GPD-FLAG-scUtp24 and integrated into pRS415-GPD-FLAG-hUTP24 by the TPCR method. All primers used are listed in S2 Table. All plasmids were confirmed by DNA sequencing.

### Yeast growth assay

The *utp24Δ*/ENP1-TAP strain supplemented with pRS416-GAL-HA-UTP24 was transformed with a pRS415-GPD-FLAG plasmid encoding wild-type or mutant Utp24. The transformed strain was grown to OD_600_ = 0.8–1.0 in 2 ml Leu- and Ura-deficient SC/Gal medium at 30 °C. After centrifugal collection and two times of wash with water, the cells were adjusted to OD_600_ = 0.2 and 5-fold serially diluted with water. The diluted cells were spotted on Ura- and Leu-deficient SC plates supplemented with galactose or glucose and incubated at 30 °C for 2 days.

### Immunoprecipitation and Western blot

Cell lysates from 200 ml culture (OD_600_ = 0.8–1) were incubated with IgG coated beads in lysis buffer (20 mM HEPES-KOH pH 8.0, 110 mM KOAc, 40 mM NaCl) for 40 min at 4°C. The immunoprecipitated proteins and total cell lysates were analyzed with SDS-PAGE and Western blot. Peroxidase-anti-peroxidase (PAP) (1:5000, Sigma), anti-Flag (1:10000, Sigma) and HRP-conjugated anti-HA antibodies (1:3000, CST) were used with appropriate dilution ratios. Anti-Krr1 polyclonal antibodies (1:2000) were raised in rabbit with recombinant Krr1 protein.

### Ribosome profiles

Ribosome profile was conducted as previously described [44]. Cell lysates of 15 OD_260_ units were separated on 7%-50% sucrose gradients.

### Mass spectrometry analysis of pre-ribosomes

Pre-ribosomes were purified as previously described [10]. The Utp24 shuffle strain was cultured in Ura-deficient SC/Gal medium and then transferred into 3 liters of YPD to grow for 14 h to OD_600_ of 1.0–2.0. The cells were collected and suspended in lysis buffer (20 mM HEPES-KOH pH 8.0, 110 mM KOAc, 40 mM NaCl) supplemented with EDTA-free protease inhibitor cocktail (Roche). The cells were broken by a tissue lyser followed by centrifugation at 6,000g for 20 min. The supernatant was incubated with IgG-coated magnetic Beaver beads (Beaverbio) for 40 min. The beads were washed three times with lysis buffer and incubated with TEV protease in lysis buffer supplemented with 5 mM DTT.

Approximately 40 μg proteins were analyzed by mass spectrometry as described [31]. The total spectral counts per 100 residues (SCPHR) were calculated for each identified protein and further normalized against UTPB proteins, yielding the relative spectral abundance factor (RSAF) (S1 Dataset) [6].

### Northern blot

Northern blot was conducted as described [10, 44]. RNAs were extracted from total cell lysates or immunoprecipitations of 2 liters culture and separated in 1.2% agarose-formaldehyde. The hybridization probes are as followed: D-A2, 5’-CGGTTTTAATTGTCCTA; A2-A3, 5’-ATGAAAACTCCACAGTG.

### Accession number

The coordinates and structural factors have been deposited into the Protein Data Bank with accession code 5YZ4.

## Supporting information

S1 Fig Pre-rRNA processing in yeast. The 35S pre-rRNA is successively processed at the A0, A1 and A2 sites to generate 20S and 27SA2 pre-rRNAs. Alternatively, 35S pre-rRNA can be first cleaved at the A3 site to yield 23S pre-rRNA, which is further cleaved at the A0, A1 and A2 sites to produce 20S pre-rRNA. The 23S pre-rRNA is also subjected to TRAMP-mediated polyadenylation and exosome-mediated degradation. 18S rRNA is produced following cleavage of 20S pre-rRNA at site D. The 27SA2 intermediate is processed into 5.8S and 25S rRNA through multiple steps. The hybridization sites of probes are labeled.
(TIF)

S1 Table Yeast strains used in this study. (PDF)

S2 Table Oligos used in this study. (PDF)

S1 Dataset Mass spectrometric data for pre-ribosomal particles. The total spectral counts per hundred residues (SCPHR) and relative spectral abundance factor (RSAF) values of identified proteins are displayed in two separate sheets. The RSAF is normalized against six UTPB proteins. The top rows include the summed SCPHR or RSAF values for 90S proteins, pre-40S proteins, pre-60S proteins, small subunit ribosomal proteins (RPS), large subunit ribosomal proteins (RPL) and total identified proteins, the total spectral counts (SpC) of reference proteins and the molar percentages of 90S and pre-40S AFs over all detected proteins.
(XLSX)
