# Supplementary figures and images for: Structural and functional analysis of Utp24, an endonuclease for processing 18S ribosomal RNA
Source: PLoS One. 2018 Apr 11;13(4):e0195723. doi: 10.1371/journal.pone.0195723 (PMC5895043; doi:10.1371/journal.pone.0195723)

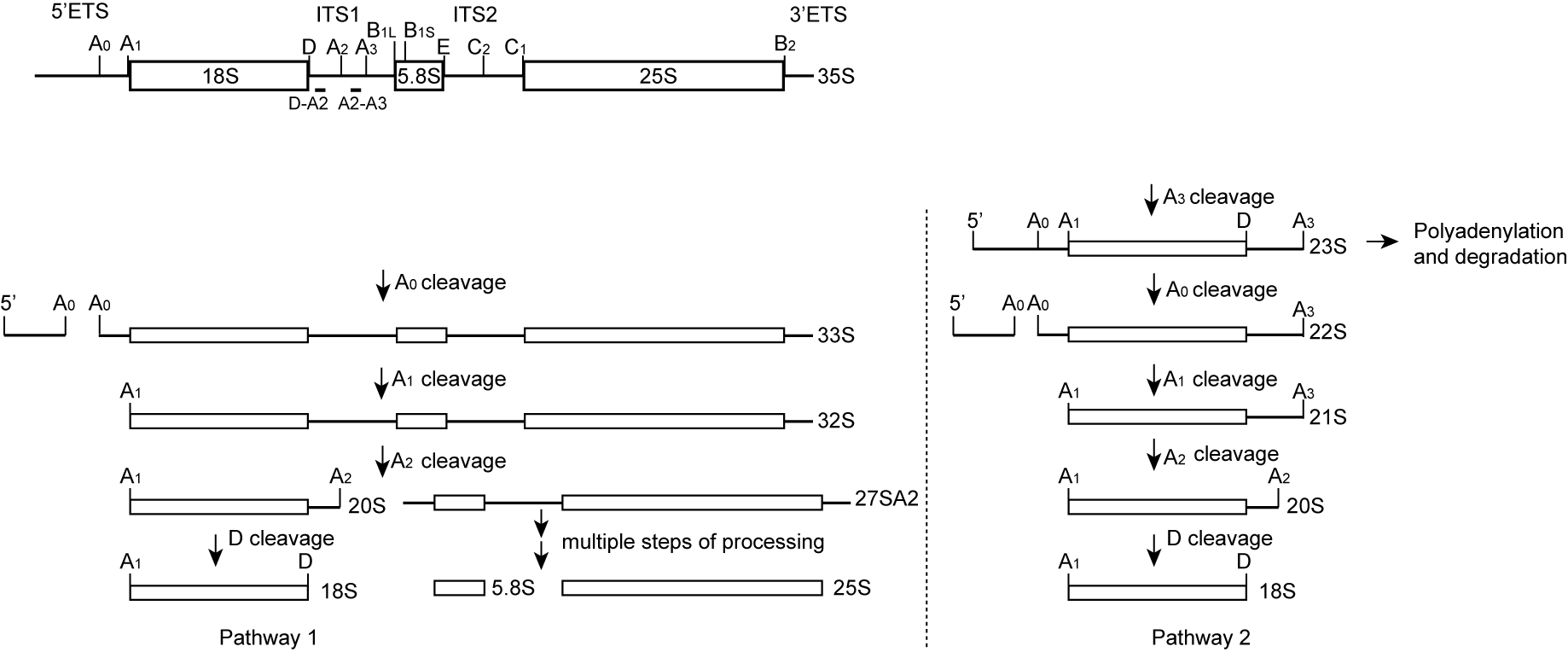

Supplement: S1 Fig — The 35S pre-rRNA is successively processed at the A0, A1 and A2 sites to generate 20S and 27SA2 pre-rRNAs. Alternatively, 35S pre-rRNA can be first cleaved at the A3 site to yield 23S pre-rRNA, which is further cleaved at the A0, A1 and A2 sites to produce 20S pre-rRNA. The 23S pre-rRNA is also subjected to TRAMP-mediated polyadenylation and exosome-mediated degradation. 18S rRNA is produced following cleavage of 20S pre-rRNA at site D. The 27SA2 intermediate is processed into 5.8S and 25S rRNA through multiple steps. The hybridization sites of probes are labeled. (TIF) [file pone.0195723.s001.tif]
