# Supplementary material for: Structural and functional analysis of Utp24, an endonuclease for processing 18S ribosomal RNA
Source: PLoS One. 2018 Apr 11;13(4):e0195723. doi: 10.1371/journal.pone.0195723 (PMC5895043; doi:10.1371/journal.pone.0195723)
Supplement: S1 Table — (PDF) [file pone.0195723.s002.pdf]

Table S1. Yeast strains used in this study

Strain	Genotype	Origin
BY4741	MATa, his3Δ1, leu2Δ0, met15Δ0, ura3Δ0	Euroscarf
ENP1-TAP	BY4741, ENP1-TAP::HIS3MX	Open Biosystems
NOC4-TAP	BY4741, NOC4-TAP::HIS3MX	Open Biosystems
Utp24 shuffle	BY4741, ENP1-TAP::HIS3MX, utp24::natNT2, [pRS416-GAL-HA-UTP24, URA3]	This study
