# Supplementary material for: Structural and functional analysis of Utp24, an endonuclease for processing 18S ribosomal RNA
Source: PLoS One. 2018 Apr 11;13(4):e0195723. doi: 10.1371/journal.pone.0195723 (PMC5895043; doi:10.1371/journal.pone.0195723)
Supplement: S2 Table — (PDF) [file pone.0195723.s003.pdf]

S2 Table. Oligos used in this study

Oligo name	Sequence(5'-3')	Usage
pET28a-spUtp24-F	CAGATTGGTGGATCCATGGGTAAAGCAAAAACTACACGCAAATTTG	In-Fusion primers for constructing pET28a-His-Smt3-spUtp24
pET28a-spUtp24-R	AGCCGGATCTCATTATCAATCCACCACATCCACCAAAC	
416-scUtp24-F	CGCTGGCACTAGTGCCGGCCGGATGGGTAAAGCTAAGAAAACAAGAAAGTTTG	TPCR primers for constructing pRS416-GAL-HA-scUtp24
416-scUtp24-R	GGAGACTTGACCAAACCTCTGGCGAAGTTAAAAGACATCTGGCAATTTTTCAATGACG	
L1	CACCTTCATGTACCTATCGTTTGTGG	Primers for deleting Utp24 gene from yeast. The 5' and 3' arms of Utp24 gene were amplified with primers L1 and L2 and L3 and L4, respectively, from yeast genomic DNA. The natNT2 cassette was amplified with overlap PCR using primers L1 and L4 and templates of pYM-N28 and the 5' and 3' arm of Utp24.
L2	CAAGGAGGGTATTCTGGGCCTCCATGTCAATTAACTGTTGTAGTATATCGATATTTCCG	
L3	GCTCTACATGAGCATGCCCTGCCCCTAAACTACTGTGTATTCACTCTTTAAGTATGTAG	
L4	CTGCATCGCGTGGAACCCATG	
415-scUtp24-F	GATGACGATGACAAGATGGGTAAAGCTAAGAAAACAAGAAAGTTTGG	In-Fusion primer for constructing pRS415-GPD-FLAG-scUtp24 and pRS415-GPD-FLAG-scUtp24 N
415-scUtp24-R	GGTATCGATAAGCTTTTAAAAGACATCTGGCAATTTTTCAATGACG	In-Fusion primer for constructing pRS415-GPD-FLAG-scUtp24 and pRS415-GPD-FLAG-scUtp24 PIN
415-scUtp24-60-F	GATGACGATGACAAGATGAAGCCACCTTATCAAGTACTGATAG	In-Fusion primer for constructing pRS415-GPD-FLAG-scUtp24 PIN
415-scUtp24-60-R	GGTATCGATAAGCTTTTACTTTATAGCTTGATTGTATTGAAAGAAAAGTGC	In-Fusion primer for constructing pRS415-GPD-FLAG-scUtp24 N
415-spUtp24-F	GATGACGATGACAAGATGGGTAAAGCAAAAACTACACGC	In-Fusion primers for constructing pRS415-GPD-FLAG-spUtp24
415-spUtp24-R	GGTATCGATAAGCTTTCAATCCACCACATCCACCAAAC	
415-hUtp24-F	GATGACGATGACAAGATGGGGAAGCAAAAGAAAACAAGG	In-Fusion primers for constructing pRS415-GPD-FLAG-hUTP24
415-hUtp24-R	GGTATCGATAAGCTTTTAGAATCGAGGGGCTCCATAATC	
415-scNhPIN-F	CATCGACTACAAGGATGACGATGACAAGATGGGTAAAGCTAAGAAAACAAGAAAGTTTG	TPCR primers for constructing pRS415-GPD-FLAG-Utp24 (scN+hPIN)
415-scNhPIN-R	GGTATCAACGAGGATGTGGTAAGGTGGCTTTATAGCTTGATTGTATTGAAAGA	

	AAAGTG	
QCscUtp24-D138N-F	CACAAGGGTACGTACGCGAATGACTG TTTAGTGCATCGAG	QuikChange primers for pRS415-GPD-FLAG-scUtp24 (D138N)
QCscUtp24-D138N-R	CTCGATGCACTAAACAGTCATTCGCG TACGTACCCTTGTG	
QCscUtp24-C101A-F	CCCGTTAATCACAGATGGTGTGATGG CAGAG	QuikChange primers for pRS415-GPD-FLAG-scUtp24 (C101A)
QCscUtp24-C101A-R	CTCTGCCATCACACCATCTGTGATTAA CGGG	
QCscUtp24-C130A-F	GAATAAAGAGACTAAGCGGTTCGCAC AAGGGTACG	QuikChange primers for pRS415-GPD-FLAG-scUtp24 (C130A)
QCscUtp24-C130A-R	CGTACCCTTGTGCGAACCGCTTAGTC TCTTTATTC	
QCscUtp24-H132A-F	GAGACTAAGCTGTTCGGCCAAGGGTA CGTACGC	QuikChange primers for pRS415-GPD-FLAG-scUtp24 (H132A)
QCscUtp24-H132A-R	GCGTACGTACCCTTGGCCGAACAGCT TAGTCTC	
QCscUtp24-C140A-F	CGTACGCGGATGACGGTTTAGTGCAT CGAG	QuikChange primers for pRS415-GPD-FLAG-scUtp24 (C140A)
QCscUtp24-C140A-R	CTCGATGCACTAAACCGTCATCCGCG TACG	
D-A2	CGGTTTTAATTGTCCTA	Northern blot probes
A2-A3	ATGAAAACTCCACAGTG	
